# Supplementary material for: Single-cell nanodroplet processing proteomics pipeline for analysis of human-derived microglia
Source: bioRxiv. 2025 Oct 4:2025.10.02.680067. Preprint. [Version 1] doi: 10.1101/2025.10.02.680067 (PMC12621868; doi:10.1101/2025.10.02.680067)
Supplement: Supplement 2 [file media-2.pdf]

Accession	Gene	GO_ID	GO_Term
P07910	HNRNPC	GO:0070935	3'-UTR-mediated mRNA stabilization
P98175	RBM10	GO:0070935	3'-UTR-mediated mRNA stabilization
Q13148	TARDBP	GO:0070935	3'-UTR-mediated mRNA stabilization
Q13151	HNRNPA0	GO:0070935	3'-UTR-mediated mRNA stabilization
Q15717	ELAVL1	GO:0070935	3'-UTR-mediated mRNA stabilization
O75489	NDUFS3	GO:0009060	aerobic respiration
O95168	NDUFB4	GO:0009060	aerobic respiration
O96000	NDUFB10	GO:0009060	aerobic respiration
P04040	CAT	GO:0009060	aerobic respiration
P14927	UQCRB	GO:0009060	aerobic respiration
P19404	NDUFV2	GO:0009060	aerobic respiration
P28331	NDUFS1	GO:0009060	aerobic respiration
P30049	ATP5F1D	GO:0009060	aerobic respiration
P31930	UQCRC1	GO:0009060	aerobic respiration
P40926	MDH2	GO:0009060	aerobic respiration
Q16718	NDUFA5	GO:0009060	aerobic respiration
O75306	NDUFS2	GO:0009060	aerobic respiration
P06576	ATP5F1B	GO:0006754	ATP biosynthetic process
P25705	ATP5F1A	GO:0006754	ATP biosynthetic process
P30049	ATP5F1D	GO:0006754	ATP biosynthetic process
P36542	ATP5F1C	GO:0006754	ATP biosynthetic process
P48047	ATP5PO	GO:0006754	ATP biosynthetic process
P56134	ATP5MF	GO:0006754	ATP biosynthetic process
P56385	ATP5ME	GO:0006754	ATP biosynthetic process
P04075	ALDOA	GO:0006754	ATP biosynthetic process
O00483	NDUFA4	GO:0045333	cellular respiration
P09669	COX6C	GO:0045333	cellular respiration
P10606	COX5B	GO:0045333	cellular respiration
P13073	COX4I1	GO:0045333	cellular respiration
P14406	COX7A2	GO:0045333	cellular respiration
P14927	UQCRB	GO:0045333	cellular respiration
P15954	COX7C	GO:0045333	cellular respiration
P20674	COX5A	GO:0045333	cellular respiration
P28331	NDUFS1	GO:0045333	cellular respiration
P31930	UQCRC1	GO:0045333	cellular respiration
P99999	CYCS	GO:0045333	cellular respiration
P0DP23;P0DP24;P0D	CALM1, CALM2, CAL	GO:0060291	long-term synaptic potentiation
P51608	MECP2	GO:0060291	long-term synaptic potentiation
Q13554	CAMK2B	GO:0060291	long-term synaptic potentiation
Q13555	CAMK2G	GO:0060291	long-term synaptic potentiation
Q9UQM7	CAMK2A	GO:0060291	long-term synaptic potentiation
P04350	TUBB4A	GO:0000226	microtubule cytoskeleton organization
P04406	GAPDH	GO:0000226	microtubule cytoskeleton organization

P07437	TUBB	GO:0000226	microtubule cytoskeleton organization
P0DPH7;P0DPH8	TUBA3C, TUBA3D	GO:0000226	microtubule cytoskeleton organization
P11137	MAP2	GO:0000226	microtubule cytoskeleton organization
P18583	SON	GO:0000226	microtubule cytoskeleton organization
P46821	MAP1B	GO:0000226	microtubule cytoskeleton organization
P68363	TUBA1B	GO:0000226	microtubule cytoskeleton organization
P68366	TUBA4A	GO:0000226	microtubule cytoskeleton organization
P68371	TUBB4B	GO:0000226	microtubule cytoskeleton organization
Q00535	CDK5	GO:0000226	microtubule cytoskeleton organization
Q13509	TUBB3	GO:0000226	microtubule cytoskeleton organization
Q13885	TUBB2A	GO:0000226	microtubule cytoskeleton organization
Q9BUF5	TUBB6	GO:0000226	microtubule cytoskeleton organization
Q9NSV4	DIAPH3	GO:0000226	microtubule cytoskeleton organization
Q9NY65	TUBA8	GO:0000226	microtubule cytoskeleton organization
Q9NZT1	CALML5	GO:0000226	microtubule cytoskeleton organization
P04350	TUBB4A	GO:0007017	microtubule-based process
P07437	TUBB	GO:0007017	microtubule-based process
P0DPH7;P0DPH8	TUBA3C, TUBA3D	GO:0007017	microtubule-based process
P68363	TUBA1B	GO:0007017	microtubule-based process
P68366	TUBA4A	GO:0007017	microtubule-based process
P68371	TUBB4B	GO:0007017	microtubule-based process
Q13509	TUBB3	GO:0007017	microtubule-based process
Q13885	TUBB2A	GO:0007017	microtubule-based process
Q9BUF5	TUBB6	GO:0007017	microtubule-based process
Q9NY65	TUBA8	GO:0007017	microtubule-based process
O00483	NDUFA4	GO:0006123	mitochondrial electron transport
P09669	COX6C	GO:0006123	mitochondrial electron transport
P10606	COX5B	GO:0006123	mitochondrial electron transport
P13073	COX4I1	GO:0006123	mitochondrial electron transport
P14406	COX7A2	GO:0006123	mitochondrial electron transport
P15954	COX7C	GO:0006123	mitochondrial electron transport
P20674	COX5A	GO:0006123	mitochondrial electron transport
P99999	CYCS	GO:0006123	mitochondrial electron transport
P04350	TUBB4A	GO:0000278	mitotic cell cycle
P07437	TUBB	GO:0000278	mitotic cell cycle
P0DPH7;P0DPH8	TUBA3C, TUBA3D	GO:0000278	mitotic cell cycle
P21127	CDK11B	GO:0000278	mitotic cell cycle
P62826	RAN	GO:0000278	mitotic cell cycle
P68363	TUBA1B	GO:0000278	mitotic cell cycle
P68366	TUBA4A	GO:0000278	mitotic cell cycle
P68371	TUBB4B	GO:0000278	mitotic cell cycle
Q00610	CLTC	GO:0000278	mitotic cell cycle
Q13123	IK	GO:0000278	mitotic cell cycle
Q13509	TUBB3	GO:0000278	mitotic cell cycle

Q13885	TUBB2A	GO:0000278	mitotic cell cycle
Q9BUF5	TUBB6	GO:0000278	mitotic cell cycle
Q9NY65	TUBA8	GO:0000278	mitotic cell cycle
Q9UQE7	SMC3	GO:0000278	mitotic cell cycle
P62714;P67775	PPP2CB, PPP2CA	GO:0000278	mitotic cell cycle
P09669	COX6C	GO:0006119	oxidative phosphorylation
P10606	COX5B	GO:0006119	oxidative phosphorylation
P13073	COX4I1	GO:0006119	oxidative phosphorylation
P14406	COX7A2	GO:0006119	oxidative phosphorylation
P14927	UQCRB	GO:0006119	oxidative phosphorylation
P15954	COX7C	GO:0006119	oxidative phosphorylation
P20674	COX5A	GO:0006119	oxidative phosphorylation
P31930	UQCRC1	GO:0006119	oxidative phosphorylation
P36542	ATP5F1C	GO:0006119	oxidative phosphorylation
P43246	MSH2	GO:0006119	oxidative phosphorylation
O75947	ATP5PD	GO:0015986	proton motive force-driven ATP synthesis
P06576	ATP5F1B	GO:0015986	proton motive force-driven ATP synthesis
P24539	ATP5PB	GO:0015986	proton motive force-driven ATP synthesis
P25705	ATP5F1A	GO:0015986	proton motive force-driven ATP synthesis
P30049	ATP5F1D	GO:0015986	proton motive force-driven ATP synthesis
P36542	ATP5F1C	GO:0015986	proton motive force-driven ATP synthesis
P48047	ATP5PO	GO:0015986	proton motive force-driven ATP synthesis
P56134	ATP5MF	GO:0015986	proton motive force-driven ATP synthesis
P56385	ATP5ME	GO:0015986	proton motive force-driven ATP synthesis
Q96IX5	ATP5MK	GO:0015986	proton motive force-driven ATP synthesis
O75494	SRSF10	GO:0048024	regulation of mRNA splicing, via spliceosome
O75525	KHDRBS3	GO:0048024	regulation of mRNA splicing, via spliceosome
P18583	SON	GO:0048024	regulation of mRNA splicing, via spliceosome
P61978	HNRNPK	GO:0048024	regulation of mRNA splicing, via spliceosome
P84103	SRSF3	GO:0048024	regulation of mRNA splicing, via spliceosome
Q07666	KHDRBS1	GO:0048024	regulation of mRNA splicing, via spliceosome
Q14498	RBM39	GO:0048024	regulation of mRNA splicing, via spliceosome
Q96MU7	YTHDC1	GO:0048024	regulation of mRNA splicing, via spliceosome
