# Supplementary material for: Single-cell nanodroplet processing proteomics pipeline for analysis of human-derived microglia
Source: bioRxiv. 2025 Oct 4:2025.10.02.680067. Preprint. [Version 1] doi: 10.1101/2025.10.02.680067 (PMC12621868; doi:10.1101/2025.10.02.680067)
Supplement: Supplement 3 [file media-3.pdf]

Accession	Gene	GO_ID	GO_Term
P00751	CFB	GO:0006956	complement activation
P01024	C3	GO:0006956	complement activation
P01031	C5	GO:0006956	complement activation
P02745	C1QA	GO:0006956	complement activation
P02748	C9	GO:0006956	complement activation
P05156	CFI	GO:0006956	complement activation
P08603	CFH	GO:0006956	complement activation
P09871	C1S	GO:0006956	complement activation
P0C0L4	C4A	GO:0006956	complement activation
P0C0L5	C4B	GO:0006956	complement activation
P10909	CLU	GO:0006956	complement activation
P13987	CD59	GO:0006956	complement activation
P00751	CFB	GO:0006957	complement activation, alternative pathway
P01024	C3	GO:0006957	complement activation, alternative pathway
P01031	C5	GO:0006957	complement activation, alternative pathway
P02748	C9	GO:0006957	complement activation, alternative pathway
P08603	CFH	GO:0006957	complement activation, alternative pathway
P01024	C3	GO:0006958	complement activation, classical pathway
P01031	C5	GO:0006958	complement activation, classical pathway
P01857	IGHG1	GO:0006958	complement activation, classical pathway
P01859	IGHG2	GO:0006958	complement activation, classical pathway
P01861	IGHG4	GO:0006958	complement activation, classical pathway
P01876	IGHA1	GO:0006958	complement activation, classical pathway
P01877	IGHA2	GO:0006958	complement activation, classical pathway
P02745	C1QA	GO:0006958	complement activation, classical pathway
P02747	C1QC	GO:0006958	complement activation, classical pathway
P02748	C9	GO:0006958	complement activation, classical pathway

P04003	C4BPA	GO:0006958	complement activation, classical pathway
P05156	CFI	GO:0006958	complement activation, classical pathway
P09871	C1S	GO:0006958	complement activation, classical pathway
P0C0L4	C4A	GO:0006958	complement activation, classical pathway
P0C0L5	C4B	GO:0006958	complement activation, classical pathway
P10909	CLU	GO:0006958	complement activation, classical pathway
Q07021	C1QBP	GO:0006958	complement activation, classical pathway
Q16181	SEPTIN7	GO:0061640	cytoskeleton-dependent cytokinesis
Q9NVA2	SEPTIN11	GO:0061640	cytoskeleton-dependent cytokinesis
Q9UHD8	SEPTIN9	GO:0061640	cytoskeleton-dependent cytokinesis
O75935	DCTN3	GO:0061640	cytoskeleton-dependent cytokinesis
P53990	IST1	GO:0061640	cytoskeleton-dependent cytokinesis
Q15019	SEPTIN2	GO:0061640	cytoskeleton-dependent cytokinesis
B5ME19;Q99613	EIF3CL, EIF3C	GO:0001732	formation of cytoplasmic translation initiation complex
O00303	EIF3F	GO:0001732	formation of cytoplasmic translation initiation complex
O15371	EIF3D	GO:0001732	formation of cytoplasmic translation initiation complex
O75821	EIF3G	GO:0001732	formation of cytoplasmic translation initiation complex
O75822	EIF3J	GO:0001732	formation of cytoplasmic translation initiation complex
P55010	EIF5	GO:0001732	formation of cytoplasmic translation initiation complex

P55884	EIF3B	GO:0001732	formation of cytoplasmic translation initiation complex
P60228	EIF3E	GO:0001732	formation of cytoplasmic translation initiation complex
Q14152	EIF3A	GO:0001732	formation of cytoplasmic translation initiation complex
Q9UBQ5	EIF3K	GO:0001732	formation of cytoplasmic translation initiation complex
Q9Y262	EIF3L	GO:0001732	formation of cytoplasmic translation initiation complex
O15372	EIF3H	GO:0001732	formation of cytoplasmic translation initiation complex
Q7L2H7	EIF3M	GO:0001732	formation of cytoplasmic translation initiation complex
O95445	APOM	GO:0034384	high-density lipoprotein particle clearance
P02647	APOA1	GO:0034384	high-density lipoprotein particle clearance
P02649	APOE	GO:0034384	high-density lipoprotein particle clearance
P02655	APOC2	GO:0034384	high-density lipoprotein particle clearance
P02652	APOA2	GO:0034384	high-density lipoprotein particle clearance
O95445	APOM	GO:0034375	high-density lipoprotein particle remodeling
P02647	APOA1	GO:0034375	high-density lipoprotein particle remodeling
P02649	APOE	GO:0034375	high-density lipoprotein particle remodeling
P02654	APOC1	GO:0034375	high-density lipoprotein particle remodeling
P02656	APOC3	GO:0034375	high-density lipoprotein particle remodeling
P06727	APOA4	GO:0034375	high-density lipoprotein particle remodeling

P02652	APOA2	GO:0034375	high-density lipoprotein particle remodeling
P10606	COX5B	GO:0006123	mitochondrial electron transport, cytochrome c to oxygen
P99999	CYCS	GO:0006123	mitochondrial electron transport, cytochrome c to oxygen
P00403	MT-CO2	GO:0006123	mitochondrial electron transport, cytochrome c to oxygen
P09669	COX6C	GO:0006123	mitochondrial electron transport, cytochrome c to oxygen
P13073	COX4I1	GO:0006123	mitochondrial electron transport, cytochrome c to oxygen
O00483	NDUFA4	GO:0006123	mitochondrial electron transport, cytochrome c to oxygen
P14406	COX7A2	GO:0006123	mitochondrial electron transport, cytochrome c to oxygen
P00734	F2	GO:0051918	negative regulation of fibrinolysis
P00747	PLG	GO:0051918	negative regulation of fibrinolysis
P02749	APOH	GO:0051918	negative regulation of fibrinolysis
P04004	VTN	GO:0051918	negative regulation of fibrinolysis
P04196	HRG	GO:0051918	negative regulation of fibrinolysis
P08697	SERPINF2	GO:0051918	negative regulation of fibrinolysis
P05121	SERPINE1	GO:0051918	negative regulation of fibrinolysis
O60220	TIMM8A	GO:0045039	protein insertion into mitochondrial inner membrane
O94826	TOMM70	GO:0045039	protein insertion into mitochondrial inner membrane

Q53H12	AGK	GO:0045039	protein insertion into mitochondrial inner membrane
Q9Y5J7	TIMM9	GO:0045039	protein insertion into mitochondrial inner membrane
Q9Y5J9	TIMM8B	GO:0045039	protein insertion into mitochondrial inner membrane
Q9Y5L4	TIMM13	GO:0045039	protein insertion into mitochondrial inner membrane
O43809	NUDT21	GO:0051262	protein tetramerization
P34897	SHMT2	GO:0051262	protein tetramerization
P04637	TP53	GO:0051262	protein tetramerization
Q15070	OXA1L	GO:0051262	protein tetramerization
Q16630	CPSF6	GO:0051262	protein tetramerization
Q8N684	CPSF7	GO:0051262	protein tetramerization
O15371	EIF3D	GO:0075525	viral translational termination-reinitiation
O75821	EIF3G	GO:0075525	viral translational termination-reinitiation
P55884	EIF3B	GO:0075525	viral translational termination-reinitiation
Q14152	EIF3A	GO:0075525	viral translational termination-reinitiation
Q9Y262	EIF3L	GO:0075525	viral translational termination-reinitiation
